# Study protocol for a randomised controlled trial of electronic cigarettes versus nicotine patch for smoking cessation

**DOI:** 10.1186/1471-2458-13-210

**Published:** 2013-03-08

**Authors:** Chris Bullen, Jonathan Williman, Colin Howe, Murray Laugesen, Hayden McRobbie, Varsha Parag, Natalie Walker

**Affiliations:** 1 National Institute for Health Innovation, School of Population Health, The University of Auckland, Private Bag 92019, Auckland 1142, New Zealand; 2 Department of Public Health and General Practice, University of Otago, Christchurch, New Zealand; 3 Health New Zealand Ltd, Lyttelton, Christchurch, New Zealand; 4 Queen Mary University of London, Wolfson Institute of Preventive Medicine, Barts and The London School of Medicine and Dentistry, Charterhouse Square, London, UK

**Keywords:** Electronic cigarettes, E-cigarettes, ENDS, Cessation, Efficacy, Safety, Randomised controlled trial, Nicotine

## Abstract

**Background:**

Electronic cigarettes (e-cigarettes or electronic nicotine delivery systems [ENDS]) are electrically powered devices generally similar in appearance to a cigarette that deliver a propylene glycol and/or glycerol mist to the airway of users when drawing on the mouthpiece. Nicotine and other substances such as flavourings may be included in the fluid vaporised by the device. People report using e-cigarettes to help quit smoking and studies of their effects on tobacco withdrawal and craving suggest good potential as smoking cessation aids. However, to date there have been no adequately powered randomised trials investigating their cessation efficacy or safety. This paper outlines the protocol for this study.

**Methods/design:**

*Design:* Parallel group, 3-arm, randomised controlled trial. *Participants:* People aged ≥18 years resident in Auckland, New Zealand (NZ) who want to quit smoking. *Intervention:* Stratified blocked randomisation to allocate participants to either Elusion™ e-cigarettes with nicotine cartridges (16 mg) or with placebo cartridges (i.e. no nicotine), or to nicotine patch (21 mg) alone. Participants randomised to the e-cigarette groups will be told to use them *ad libitum* for one week before and 12 weeks after quit day, while participants randomised to patches will be told to use them daily for the same period. All participants will be offered behavioural support to quit from the NZ Quitline. *Primary* o*utcome:* Biochemically verified (exhaled carbon monoxide) continuous abstinence at six months after quit day. *Sample size:* 657 people (292 in both the nicotine e-cigarette and nicotine patch groups and 73 in the placebo e-cigarettes group) will provide 80% power at *p* = 0.05 to detect an absolute difference of 10% in abstinence between the nicotine e-cigarette and nicotine patch groups, and 15% between the nicotine and placebo e-cigarette groups.

**Discussion:**

This trial will inform international debate and policy on the regulation and availability of e-cigarettes. If shown to be efficacious and safe, these devices could help many smokers as an alternative smoking cessation aid to standard nicotine products.

**Trial registration:**

Australian NZ Clinical Trials Registry (ACTRN12610000866000).

## Background

Most smokers want to quit and make attempts to do so but the majority of these attempts fail largely because of their dependence on nicotine and non-nicotine sensory and behavioural cues that reinforce their smoking behaviour [1]. Psychological and pharmacological smoking cessation treatments help smokers quit and are one of the most cost-effective health interventions available [2]. Indeed, nicotine replacement therapy (NRT) almost doubles quit rates irrespective of the level of additional behavioural support [3]. Nevertheless, absolute long-term quit rates with NRT are low: typically, fewer than 20% of people quitting with NRT plus behavioural support are still abstinent at 12 months [3]. Even the most rapid-acting and highest strength NRT products do not deliver nicotine in doses or at the same speed as cigarettes [4]. With the possible exception of the nicotine inhalator, neither do they replace the sensory and behavioural cues associated with cigarette use. Indeed, many smokers find available NRTs unhelpful, unpleasant or difficult to use and want more product choice [5].

Electronic nicotine delivery systems (ENDS), more commonly known as electronic cigarettes (hereafter abbreviated to 'e-cigarettes' or 'e-cigs'), are so-called because of their physical resemblance to a standard tobacco cigarette and their electronic vapour-generating mechanism. In 2004 a Beijing-based company, Ruyan Group (Holdings) Ltd, patented and launched the first of these devices [6]. Many other manufacturers have since made similar devices. All contain a mouthpiece, a micro-electrical circuit, a vaporiser, and a rechargeable lithium ion battery. The solution in replaceable cartridges or used to fill a reservoir in some models typically contains a solution of humectants (e.g. propylene glycol and/or glycerol), and nicotine and flavourings. When a user draws air through the e-cigarette the micro-electrical circuit activates an electric coil to heat and vaporise a small amount of the nicotine solution, creating a visible cloud of mist that may be inhaled by the user [7].

It has been hypothesised that e-cigarettes could be more effective than NRT at helping people quit smoking by delivering nicotine together with mimicking the physical, sensory and behavioural aspects of cigarette use [1,8]. In international online surveys one of the key reasons people report buying e-cigarettes is to help quit smoking [9,10].

In 2008 we undertook the first study of the effect of the e-cigarettes on desire to smoke and withdrawal symptoms and found that they were as effective as the NRT inhaler on reducing withdrawal but more pleasant and acceptable to use [8]. Further research has generally supported these early findings, and none to date have found evidence of harm [6]. We also found that the speed of nicotine delivery and serum levels obtained was substantially less than tobacco cigarettes. This has been corroborated with other brands by other research groups [11,12].

A related question is the extent to which any quitting assistance provided by e-cigarettes might be attributable simply to the behavioural replacement they provide, as suggested by a study of a nicotine-free inhaler device by Capponeto et al. [13]. This is relevant to countries such as New Zealand where e-cigarettes are available for sale over the counter but without cartridges containing nicotine.

E-cigarette sales are increasing rapidly: in the US alone there are an estimated 2.5 million users with sales of $300 million a year [14]. Internet search volumes have surpassed search volumes for both NRT and varenicline in the UK, US and Canada [15]. Despite this evidence of consumer demand, there are mixed views in the tobacco control community as to what role, if any, e-cigarettes might play in helping smokers to cease tobacco use. Strong concerns have been expressed over their potential to be a gateway to tobacco smoking, the safety of the inhaled vapours and ability of the devices to deliver nicotine as claimed [16-18].

Although a few studies have been conducted that show promise for the potential of e-cigarettes as cessation aids [19-21], none have been adequately powered. As noted, a number of surveys of users suggest potential in this area, but to date, no randomised trials that evaluate the long- term efficacy e-cigarettes on abstinence, in smokers motivated to quit, have been completed. Nor has there been a systematic evaluation of their safety. A well-designed trial is urgently needed to inform policy development on the regulation and availability of these and the many other emerging nicotine cigarette substitute products [22,23].

## Aim

The primary aim of the trial is to assess the effectiveness, acceptability, patterns of use and safety of e-cigarettes as a smoking cessation aid. The study will determine the quit rates of smokers using nicotine versus nicotine patch and versus placebo (non-nicotine) e-cigarettes at six months. The trial hypotheses are:

• nicotine e-cigarettes are more effective than nicotine patches on smoking abstinence at six months;

• nicotine e-cigarettes are more effective than placebo e-cigarettes on smoking abstinence at six months;

• nicotine e-cigarettes are more effective than placebo e-cigarettes at relieving withdrawal symptoms and suppressing cravings to smoke;

• e-cigarettes are safe to use over a 13 week period;

• e-cigarettes are an acceptable treatment for smokers trying to quit.

## Methods/design

### Design

A three-arm, parallel-group randomised controlled trial.

### Study population

People from throughout Auckland, New Zealand, who smoke tobacco cigarettes and are motivated to quit.

### Inclusion and exclusion criteria

Participants will be eligible provided they are at least 18 years of age, have smoked at least 10 cigarettes a day for the past year, want to stop smoking, are able to provide verbal consent and have access to a telephone. Pregnant and breastfeeding women will be excluded, as will be people using other smoking cessation medications (including other forms of NRT, bupropion, clonidine, nortriptyline or varenicline) or who are clients of a smoking cessation programme or enrolled in another trial. People will be excluded if they report having had a heart attack, stroke or severe angina in the previous two weeks; poorly controlled asthma or other airways disease from self-report; poorly controlled diabetes mellitus; severe allergies; poorly controlled psychiatric disorders or current chemical dependence other than that involving nicotine.

### Recruitment

People resident in the Auckland region will be informed of the trial through short articles about the study in free weekly community newspapers and targeted television programming to patients waiting to see their doctor in the waiting rooms of community general practices. People interested in enrolling will be invited to contact the study centre at the University of Auckland’s National Institute for Health Innovation (NIHI) by free telephone to obtain further information, complete informed consent and undergo eligibility pre-screening. We hope to recruit as many Māori (indigenous New Zealanders) as possible to be able to test any effect of the intervention on Māori compared with non-Māori participants, because 40% of Māori smoke tobacco and have a high burden of smoking-related illness.

### Randomisation, allocation concealment and sequence generation

Callers will be told about the trial and asked if they would like to take part by a research assistant. If interested, they will be asked for basic demographic data (age, sex and ethnicity) and checked to see if they meet the trial inclusion criteria. Those who provide further details about their level of nicotine dependence (determined by the Fagerström Test for Nicotine Dependence [FTND] questionnaire [24]) will be randomised by a central computer programme to one of the three study groups using stratified block randomisation with block sizes of nine. The randomisation sequence will be prepared in advance by the study statistician (VP) but will be concealed from research assistants enrolling participants. Participants will be assigned to the 16 mg e-cigarette and nicotine patch groups in a ratio of 1:1 (290 in each) and to the nicotine e-cigarette and placebo e-cigarette groups in a ratio of 4:1 (73 in the latter arm). Three stratification factors will be used: ethnicity (Māori, Pacific, non-Māori non-Pacific), sex (male/female) and level of nicotine dependence (> 5 or ≤ 5 on the FTND [24]).

### Blinding

Due to the nature of the treatment it is not possible to blind participants to the use of e-cigarettes or nicotine patch. However, participants will be blind to the allocation of nicotine or placebo e-cigarettes as there is no difference in appearance or odour of the mist emitted from the 16 mg/ml nicotine and 0 mg/ml nicotine cartridges, nor do they differ in physical appearance. Members of the trial steering committee, management committee, and other team members (with the exception of the project co-ordinator and research assistants) will remain blind to treatment allocation until the code is broken after the last follow-up call is completed and the data recorded. The project co-ordinator and research assistants are not blinded as they are responsible for distributing the treatment to participants. To minimise the risk of bias, strict protocols for follow-up assessment procedures will be developed and the research assistants trained in adhering to these.

### Study interventions

Participants will be randomly allocated to Elusion™ e-cigarettes supplied by PGM International with nicotine cartridges (16 mg), to nicotine patch (Habitrol^®^ 21 mg patch, distributed in NZ by Novartis Consumer Health Australasia Pty. Ltd.) or to e-cigarettes with placebo (0 mg) cartridges in a ratio of 4:4:1. Participants issued with an e-cigarette will be instructed to charge the batteries daily for reliable operation using a USB charger that comes with the device, alone or in combination (if needed) with an adaptor plug.

Participants in the 16 mg nicotine e-cigarette treatment arm will be sent (by courier) a free e-cigarette and sufficient nicotine cartridges (16 mg/ml) to last for four to five weeks. Participants will be instructed to use the device *ad libitum* one week before their quit day to familiarise themselves with its operation and on their designated quit day will stop smoking tobacco cigarettes and instead use the e-cigarette exclusively for the next 12 weeks.

Those in the placebo e-cigarette arm will be sent a free e-cigarette and cartridges containing 0 mg nicotine. Participants will use the device in the same manner as described above for the nicotine e-cigarette treatment group. The cartridges will be labelled in such a way that participants cannot detect if they contain nicotine.

In the nicotine patch arm, participants will be sent (by post) vouchers to be exchanged for a minimal dispensing fee (NZ$3) at any community pharmacy for 21 mg (full strength, 24 hour) nicotine patches. All participants will receive a $5 voucher to be used at any community pharmacy on any product they wish, to cover their out of pocket expenses. Participants will use the nicotine patch daily for one week before their quit day to familiarise themselves with its use. On their designated quit day they will stop smoking and use nicotine patches daily for the next 12 weeks. This dosing regimen is consistent with what is now regarded as standard treatment in NZ and many other countries. The 21 mg strength patch is likely to be sufficient nicotine replacement for the majority of smokers recruited into the trial.

**Figure 1:**
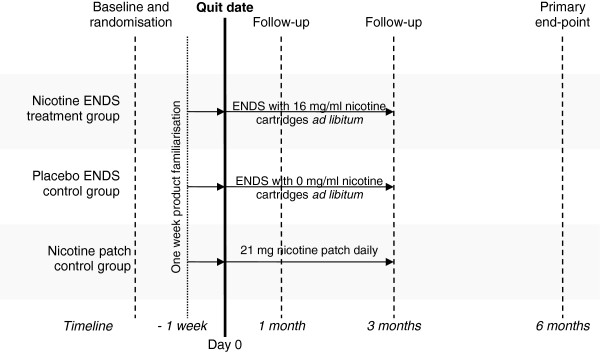
Trial schema showing key design features and timelines.

All participants will be referred to the NZ toll-free smoking cessation support helpline, Quitline (http://www.quit.org.nz), for a standardised cessation behavioural support programme delivered by trained advisors, with a minimum of one follow-up support telephone call over 8–12 weeks. If people do not want to receive this support, they will be able to access other Quitline support services such as ‘Txt2Quit’ (a mobile phone based support service), ‘QuitCoach’ (an internet based support service), and the ‘Quitter’s community’ (an internet based blogging forum) (Figure 1).

### Baseline assessments

Following randomisation the following additional baseline data will be collected from each participant: *Demographic information*: socio-economic position (based on education level attained); *Smoking history*: age when started smoking, number of cigarettes smoked per day, number of years as regular smoker, type of cigarettes smoked per day (e.g. roll-your-own or factory-made), usual pouch/pack size (and how long a pack lasts), number of previous unsuccessful attempts to give up in past 12 months and the method used including past history of any e-cigarette use; *Other smoking related information:* self-rated chances of quitting, other household members smoking at home or in cars with participant; *Use of any other smoking cessation treatments*: use of NRT or non-nicotine methods of cessation; *Physical signs and symptoms associated with withdrawal*: measured using the Autonomy Over Smoking Scale (AUTOS) [25]; *Chances of quitting: self-rated chances of quitting success measured on a scale of 1–5*; *Concomitant medication*: information about types of medication currently used; *Behavioural factors in smoking dependence*: measured using the Glover-Nilsson Smoking Behavioural Questionnaire (GH-SBQ) [26]. A total GN-SBQ score is calculated by summing all 11 items (range 0 to 4) with a score < 12 indicating a mild level of behavioural dependence, 12–22 a moderate level of behavioural dependence, 23–33 a strong level of behavioural dependence and >33 a very strong level of behavioural dependence.

### Primary outcome

The primary outcome for the trial is the proportion of participants who maintain sustained (continuous) abstinence from smoking for six months after their quit day. Abstinence is defined using the Russell Standard (i.e. intention to treat analysis, smoking five cigarettes in all [from the Quit date], plus biochemical verification of self-reported abstinence via exhaled carbon monoxide [CO] measurement, using the Bedfont Microplus device, of less than 10 parts per million) [27].

### Secondary outcomes

The following secondary outcome measures are being assessed in all participants at quit day and at one, three and six months after the Quit date: *Continuous abstinence* (as defined, at one and three months); *Seven-day point prevalence*: defined as the proportion of participants who self-report having smoked no cigarettes (not even a puff) in the past 7 days; *Use of cigarettes*: smoking at any time-point will trigger the following assessments - number of cigarettes smoked per day; proportion of participants who have significantly reduced daily smoking (defined as reducing consumption by at least 25% in terms of numbers of cigarettes per day or weight of loose tobacco per day); *Participants’ treatment compliance:* number of patches or e-cigarette cartridges used per day; early stopping and reasons why; *Perception of the product*: participant views on using e-cigarettes or patches as a smoking cessation aid (including what they liked and disliked about it and whether they would recommend the treatment to another smoker); *Use of any other smoking cessation treatments*; *Physical signs and symptoms associated with withdrawal*; *Pre-quitting stage of addiction and smoking latency: using a scale for latency to smoking developed by Ursprung* et al. [28]*. Adverse events*: Information regarding any adverse events and whether they are related to treatment.

### Sample size

A sample size of 657 people (292 in both nicotine-cigarette and nicotine patch groups and 73 in the placebo e-cigarette group) will provide 80% power at a two-sided p = 0.05 to detect an absolute difference of 10% in smoking abstinence rates between the nicotine e-cigarette and patch groups, and a difference of 15% between the nicotine and placebo e-cigarette groups. This assumes quit rates of 15% in the placebo e-cigarette group and 20% in the nicotine patch group (based on meta-analyses of NRT trials by Fiore et al. [29] and our own trials of nicotine patch [30]). This sample size allows for analysis of consistency of effects for pre-specified subgroups (e.g. Maori, non-Maori) to be assessed. Based on our previous experience we estimate recruitment will take approximately 12–15 months [31].

### Withdrawal criteria

Should participants require discontinuation of study treatment for any reason, (such as significant intolerance to the study treatment, any serious clinical adverse event, inter-current illness, pregnancy or other condition that indicates to the principal investigator that continued treatment is not in their best interest) or if they elect to cease taking treatment, follow-up calls and data collection will continue as scheduled as if they were continuing with the randomised treatment. If the participant discontinues treatment due to a serious adverse event, the participant will be followed until the event resolves or there is a return to a clinically acceptable medical status. Participant deaths or serious adverse events within 30 days of discontinuation will be reported to the project co-ordinator.

### Data management

We will capture all study data on a web-based data management system using an Oracle database. Validation rules for each case record form have been pre-specified and include range checks so inaccuracies in data collection can be identified early. A query is raised for values outside the allowed range or if data are missing, and the form amended as soon as a query is resolved.

### Data monitoring

An independent study monitor will perform audits of every randomised participant’s record and ensure that study documentation is up-to-date, record keeping adheres to the study protocol and with regulatory requirements, and handling of the study medication is appropriate.

### Statistical analysis

A senior biostatistician determined the sample size and wrote the statistical analysis plan agreed upon by all members of the Steering Committee specifying *a priori* all analyses to be undertaken. All statistical analyses will be performed using SAS version 9.3 (SAS Institute Inc. Cary, North Carolina, US) and R version 2.11.1 software (http://www.r-project.org). All regression analyses will be conducted for the following two comparisons: 16 mg e-cigarette versus nicotine patch groups; and 16 mg e-cigarette versus 0 mg e-cigarette groups.

No interim analyses will be undertaken. All tests of significance will be two-tailed. The primary analyses will be carried out on an intention to treat (ITT) basis. The ITT population will comprise all randomised participants regardless of whether they actually satisfied the entry criteria, the treatment was actually received and they subsequent withdrew or deviated from the protocol. The numbers discontinuing treatment prematurely for any reason will be summarised by treatment group and by reasons for discontinuation. The incidence of all suspected serious adverse treatment reactions will be summarised by treatment group in line with the CONSORT 2010 recommendations [32]. Simple incidence rates, relative and absolute risks, numbers needed to treat will be calculated for all binary variables, and the treatment groups will be compared using chi-squared tests with multiple logistic regression analysis adjusting for other variables as appropriate. The distribution of all continuous outcomes will be assessed for normality and skewed data will be subjected to an appropriate transformation before analysis. The change from baseline in physical signs and symptoms associated with withdrawal (AUTOS) [25] and number of cigarettes smoked per day will be analysed using repeated measures models adjusting for baseline value. The proportion of participants who have significantly reduced their daily smoking level (defined as reducing consumption by at least 50% in terms of numbers of cigarettes per day) will be calculated, and in users of loose tobacco, the size of pouch/package currently bought and number of days taken to smoke its contents measured. Finally, the number of other quit attempts in the last six months will be calculated.

A per-protocol analysis will be performed for the primary analysis to check the robustness of the results where participants with any major protocol violations such as cross-over treatments, withdrawals and loss to follow-up will be excluded. Secondary analyses will be conducted with overall cessation rates corrected for discordance between reported and biochemically confirmed cessation. The consistency of effects for pre-specified sub-groups will also be assessed using tests for heterogeneity. Time-to-first-lapse will be analysed using Kaplan-Meier curves, the log rank test and Cox proportional hazards regression analysis.

### Cost effectiveness analysis

If the primary outcome of the trial is positive then cost analyses will be undertaken. Cost outcomes will include cost per quitter, cost per person reducing their daily cigarette consumption and incremental cost-effectiveness ratio. These data will then be compared with data from the NZ Quitline and other cessation service providers, in addition to information from various international studies, using a health sector perspective. The tobacco expenditure savings to smokers who quit or cut down will also be calculated, especially to low-income smokers, using data on the daily amount smoked prior to quitting and the price of the products smoked. For those who cut down daily consumption by 25% or more, the cost savings per person reducing their daily cigarette consumption will be calculated.

### Ethical considerations

Ethics approval was granted from the New Zealand Ministry of Health’s Northern Ethics Committee (Ethics number NTX/10/11/111). Verbal consent will be obtained at the time of contact with the research team. However, a written consent form and patient information sheet will be posted to participants for their information, requesting that they send it back to the study centre in a pre-paid envelope, once signed. All data will be entered, stored and backed-up in a secure manner via a password-protected data management system. Participants will be acknowledged in all publications and presentation of the results.

## Discussion

There are many challenges with the design of this study. A key issue is that of intervention product selection. There is a large and growing number and diversity of e-cigarette products on the market, with only limited evidence available on their performance and quality. E-cigarettes differ in their characteristics such as the ability to deliver vapour reliably, the concentration of nicotine in the solution, flavour and other additives, and amount of vapour produced [31]. These differences may impact on the efficacy and acceptability of a particular brand or model so it is possible that different trial findings may be obtained using other brands or models of e-cigarette. Furthermore, e-cigarettes are evolving rapidly and a brand tested in a trial may no longer be on the market by the time the study is completed and results published.

We chose to use a particular brand (Elusion™) and model of e-cigarette for three reasons: its wide availability and popularity in New Zealand and Australia in 2011; we were able to obtain good evidence of the quality of cartridge solution production; and it was made available to us at no cost by the distributor without restrictions.

The choice of a ‘usual care’ comparator was not straightforward. We elected to use the 21 mg nicotine patch, by far the most widely used NRT product in New Zealand. Patch use will also enable us to assess whether any excess of adverse outcomes is due to the e-cigarette. The alternative comparator most seriously considered was the medicinal nicotine inhaler because of its similar use characteristics to e-cigarettes (oral inhalation, on demand use, use of hands to apply). However, it is not subsidised in NZ and to include it would have added substantial cost; also its use is aversive to start with and so a barrier for many people [33]. We chose a three-arm design with a placebo e-cigarette arm to answer the question of whether any observed efficacy could be due to behavioural replacement alone.

Users report needing time to familiarise themselves with e-cigarettes to get satisfaction [21,32], and studies that have reported some of the highest blood nicotine levels associated with e-cigarette use were undertaken in experienced users [33]. We have attempted to address this by providing participants with detailed illustrated instructions of how to use e-cigarettes, and requiring that they try them for a week prior to embarking on their quitting attempt.

The trial will report on clinical efficacy and adverse effects over a six month period of observation. This follow-up period is sufficient to determine if smokers are assisted to quit and to see if the use of nicotine e-cigarettes leads to an excess of adverse effects after eight week’s use over and above placebo e-cigarettes or nicotine patch. The results will make a valuable contribution to the Cochrane Systematic Review on electronic cigarettes for smoking cessation and reduction [34].

The study does not address the safety of long-term use of e-cigarettes (beyond 13 weeks). Neither will the study address the role of e-cigarettes as a ‘gateway’ product to tobacco smoking or a trigger to relapse to tobacco smoking. To answer these questions will require other studies using different designs.

Finally, our study will not provide a definitive answer to address all e-cigarette regulation issues. However, should the findings be in favour of nicotine e-cigarettes without additional adverse effects then it will establish a new balance between clinical benefit and harm for three month’s use of e-cigarettes.

## Abbreviations

FTND: Fagerström test for nicotine dependence; NIHI: National Institute for Health Innovation; NZ: New Zealand.

## Competing interests

The e-cigarettes used in this trial were provided by PGM International Ltd, NZ. The nicotine patches used by study participants are 21 mg strength Habitrol^®^ brand supplied by Novartis, obtained from community pharmacies at a heavily subsidised price using ‘Quit Cards’ (exchange vouchers) dispensed by registered healthcare providers. Neither PGM International Ltd nor Novartis had any role in the study design, data collection, data analysis, data interpretation, or writing of this publication. All authors declare that (1) no authors have received support from any companies for the submitted work; (2) CB and HM have previously undertaken research on behalf of NicoNovum, but prior to the purchase of the company by RJ Reynolds. CB, ML and HM previously undertook research for Ruyan (an e-cigarette manufacturer). HM has received honoraria for speaking at research symposia and received benefits in kind and travel support from, and has provided consultancy to the manufacturers of smoking cessation medications. NW has provided consultancy to the manufacturers of smoking cessation medications, received honoraria for speaking at a research meeting and received benefits in kind and travel support from a manufacturer of smoking cessation medications. All authors, with the exception of JW, were involved in a previous trial investigating the effect of reduced nicotine cigarettes on smoking cessation. This previous trial involved the use of Quest 3 cigarettes purchased from Vector Group Ltd, USA. The tobacco company had no role in development of the study design, data collection, data analysis, data interpretation, or writing of any of the trial publications; (3) their spouses, partners, or children have no financial relationships that may be relevant to the submitted work; and (4) all authors have no non-financial interests that may be relevant to the submitted work.

## Authors’ contributions

CB, NW, HM and ML conceived the original idea for the trial, sought and obtained funding CB, NW, HM, ML, CH, VP and JW wrote the study protocol. CH manages the day to day running of the trial, including all participant follow-up. VP will undertake all data analyses. This protocol paper was written by CB and JW with input from all co-authors. CB is guarantor for this paper. All authors read and approved the final manuscript.

## Current status

The trial started recruiting participants in August 2011. Trial findings are likely to be available in September 2013.

## Pre-publication history

The pre-publication history for this paper can be accessed here:

http://www.biomedcentral.com/1471-2458/13/210/prepub
